# Diffuse Axonal Injury at Ultra-High Field MRI

**DOI:** 10.1371/journal.pone.0122329

**Published:** 2015-03-20

**Authors:** Christoph Moenninghoff, Oliver Kraff, Stefan Maderwald, Lale Umutlu, Jens M. Theysohn, Adrian Ringelstein, Karsten H. Wrede, Cornelius Deuschl, Jan Altmeppen, Mark E. Ladd, Michael Forsting, Harald H. Quick, Marc Schlamann

**Affiliations:** 1 Institute of Diagnostic and Interventional Radiology and Neuroradiology, University Hospital Essen, Essen, Germany; 2 Erwin L. Hahn Institute for Magnetic Resonance Imaging, University Duisburg-Essen, Essen, Germany; 3 Department of Neurosurgery, University Hospital Essen, Essen, Germany; 4 Division of Medical Physics in Radiology, German Cancer Research Center (DKFZ), Heidelberg, Germany; 5 High Field and Hybrid MR Imaging, University Hospital Essen, Essen, Germany; 6 Institute for Neuroradiology, University Hospital Giessen, Giessen, Germany; University Zurich, SWITZERLAND

## Abstract

**Objective:**

Diffuse axonal injury (DAI) is a specific type of traumatic brain injury caused by shearing forces leading to widespread tearing of axons and small vessels. Traumatic microbleeds (TMBs) are regarded as a radiological marker for DAI. This study aims to compare DAI-associated TMBs at 3 Tesla (T) and 7 T susceptibility weighted imaging (SWI) to evaluate possible diagnostic benefits of ultra-high field (UHF) MRI.

**Material and Methods:**

10 study participants (4 male, 6 female, age range 20-74 years) with known DAI were included. All MR exams were performed with a 3 T MR system (Magnetom Skyra) and a 7 T MR research system (Magnetom 7 T, Siemens AG, Healthcare Sector, Erlangen, Germany) each in combination with a 32-channel-receive coil. The average time interval between trauma and imaging was 22 months. Location and count of TMBs were independently evaluated by two neuroradiologists on 3 T and 7 T SWI images with similar and additionally increased spatial resolution at 7 T. Inter- and intraobserver reliability was assessed using the interclass correlation coefficient (ICC). Count and diameter of TMB were evaluated with Wilcoxon signed rank test.

**Results:**

Susceptibility weighted imaging revealed a total of 485 TMBs (range 1-190, median 25) at 3 T, 584 TMBs (plus 20%, range 1-262, median 30.5) at 7 T with similar spatial resolution, and 684 TMBs (plus 41%, range 1-288, median 39.5) at 7 T with 10-times higher spatial resolution. Hemorrhagic DAI appeared significantly larger at 7 T compared to 3 T (p = 0.005). Inter- and intraobserver correlation regarding the counted TMB was high and almost equal 3 T and 7 T.

**Conclusion:**

7 T SWI improves the depiction of small hemorrhagic DAI compared to 3 T and may be supplementary to lower field strengths for diagnostic in inconclusive or medicolegal cases.

## Introduction

Traumatic brain injury (TBI) is a major cause of death in the population between 15 and 40 years of age in the industrialized nations. The incidence of TBI is estimated to be 200/100.000 inhabitants in closed head traumas and 12/100.000 inhabitants in penetrating head traumas [1]. Diffuse axonal injury (DAI) can be found in 72% of patients with moderate or severe head injury and is a major reason for morbidity and neurological disorders in the chronic phase of TBI [2–4]. First described in 1956, DAI are defined as small brain lesions of less than 15 mm maximum diameter located in areas of grey-white matter junction and midline structures, which are vulnerable to shear forces [5,6]. DAI is based on a widespread damage of axons and small vessels in the white matter of the brain caused by acceleration-deceleration effects and rotational forces in head trauma [5]. Per definition DAI is known as progression from disruption in axonal transport followed by axonal swelling and secondary disconnection, finally leading to Wallerian degeneration. This axonal degeneration is not only limited to the acute and subacute phase of TBI, but may be followed by a progressive, long-term neurodegeneration with disconnection of brain networks [7–9].

Three grades of DAI, which correlate with neurological impairment, are differentiated by histopathological findings: In grade 1 histological evidence of DAI is found in the white matter of the cerebral hemispheres; in grade 2 additional focal lesions in the corpus callosum can be detected, and in grade 3 further brain stem lesions are present [5]. Traumatic microbleeds (TMBs) in the white matter are considered as radiological marker for DAI [10]. Magnetic resonance imaging (MRI) can visualize non-hemorrhagic white matter lesions related to DAI, which are caused by axonal damage with local edema in the acute phase and Wallerian degeneration in the chronic phase. Computed tomography underestimates the amount of DAI compared with MRI [11]. Microbleeds are paramagnetic and induce local inhomogeneity of the magnetic field resulting in a fast decay of the local T2*-weighted (w) MRI signal intensity. Hence, MRI evolved as the imaging modality of choice to detect hemorrhagic and non-hemorrhagic tissue damages related to DAI [10,12–14]. The sensitivity for susceptibility contrast increases with the magnetic field strength in an almost linear manner [10]. 3 Tesla (T) T2*w gradient echo (GRE) sequences have been found to be twice as sensitive as 1.5 T MRI in the assessment of DAI [10,13]. Susceptibility weighted imaging (SWI) is an MRI technique based on a fully flow compensated, long echo, 3D gradient recalled echo (GRE) pulse sequence. It combines filtered magnitude and phase data to produce an enhanced contrast magnitude image, which shows susceptibility differences between tissues e.g. microhemorrhages, venous vasculature and calcifications [15]. It is more sensitive to cerebral microhemorrhages than T2*w GRE sequences, especially when combined with high magnetic field strengths [16–18]. The depiction of cerebral microbleeds can also be influenced by choice of echo time (TE) and spatial resolution: Longer TE leads to more dephasing and increases the susceptibility effect [19]. Higher spatial resolution reduces partial volume effects and allows the depiction of smaller cerebral microbleeds [20]. In clinical practice 1.5 T MRI is regarded to be sufficient for the detection of DAI-related TMBs, but MRI at higher field strength is known to be more sensitive [10]. An improved depiction of small hemorrhagic DAI at 7 T may be important for patients with post-concussive syndrome and closed head trauma. These patients often suffer from posttraumatic cognitive impairment, even though 1.5 T or 3 T MRI do not present pathological findings, resulting in misdiagnosis and in contradictory medical assessment [3].

We hypothesize that 7 T SWI is beneficial for the visualization of hemorrhagic DAI compared to 3 T due to synergistic effects of susceptibility and higher achievable spatial resolution at 7 T.

The purpose of this study was to compare the number and size of DAI-associated TMB at 3 T and 7 T with SWI with assessment of the intra- and interrater reliability.

## Materials and Methods

### Patients

Between July 2012 and April 2014 ten (4 male, 6 female) patients with DAI confirmed by 1.5 T MRI or CT and clinical history were prospectively included. The age of the study participants ranged from 20 to 74 years (mean age 42 years, standard deviation 19.8 years). Persons younger than 18 years and those with general contraindications against MRI examinations (e.g. pacemaker, metallic implants with unknown compatibility at 7 T, claustrophobia, neurological disease other than TBI) were excluded from the study.

Detailed characteristics of the study participants are given in Table 1.

**Table 1:** Clinical and demographic data of 10 DAI patients.

Patient number	Sex	Age	Initial GCS score	DAI grade (MRI)	Coma duration (days)	MRI after trauma (months)	GOS	Kind of trauma
1	m	20	4	III	2	45	GR	TA (cyclist)
2	f	23	5	III	5	38	SD	fall (equestrian)
3	f	39	7	I	3	96	MD	fall (6 m height)
4	f	74	13	I	0.25	6	MD	TA (pedestrian)
5	m	44	8	III	0.5	240	GR	TA (cyclist)
6	f	57	10	I	1	26	GR	fall (equestrian)
7	f	25	12	II	4	11	SD	TA (car driver)
8	f	20	5	III	21	14	GR	TA (car driver)
9	m	61	9	I	0.5	17	GR	fall (4 m height)
10	m	59	5	I	1	3	MD	TA (cyclist)

Abbreviations: GCS = Glasgow coma scale; GOS = Glasgow outcome scale; GR = good recovery; f = female;

m = male; MD = mild disability; MRI = magnetic resonance imaging; N/A = not available; SD = severe disability; TA = traffic accident

The included patients had no history of stroke, diabetes mellitus, chronic alcohol abuse, and anticoagulant or antiplatelet therapy as possible causes of cerebral microbleeds. The mean time elapsing between TBI and MRI examinations was 22 months (range 3–240, standard deviation 72 months). The study was conducted in conformance with the Declaration of Helsinki and approved by the Ethics Commission of the Medical Faculty of the University Duisburg-Essen (study number 11–4898-BO). Written informed consent was obtained from each volunteer before the examination.

### MRI examinations

Each study participant underwent two MRI examinations. The scan order—3 T followed by 7 T was performed in 9 of 10 patients within one week. Patient 10 was examined at both field strengths with a delay of 8 weeks. Ultra-high field (UHF) MR examinations were performed on a 7 T whole-body research system (Magnetom 7 T, Siemens AG, Healthcare Sector, Erlangen, Germany). All examinations at 3 T were performed on a high-end clinical MR system (Magnetom Skyra, Siemens AG, Healthcare Sector, Erlangen, Germany). Both MR systems were used in combination with 32-channel radiofrequency (RF) head coils (3 T: receive only. Siemens, Erlangen, Germany; 7 T: transmit/receive. Nova Medical, New York, USA). The multichannel RF coils allowed for parallel imaging techniques at both field strengths (GRAPPA, acceleration factor of R = 2).

At 3 T, transversal SWI was acquired with sequence parameters as provided by the vendor (Table 2).

**Table 2:** MR sequence parameters.

SWI sequence parameters	3T	7T	7T HR
TR [ms]	28	29	29
TE [ms]	20	15	15
flip angle [°]	15	15	15
bandwidth [Hz/pixel]	120	160	160
matrix [Base x Phase]	320 x 272	320 x 320	896 x 896
acquired voxel size [mm^3^]	0.70x0.80x2.6	0.70x0.70x2.0	0.25x0.25x1.5
interpolated voxel size [mm^3^]	0.70x0.70x2.0	0.70x0.70x2.0	0.25x0.25x1.5
number of slices	80	80	104
GRAPPA factor (reference lines)	2 (24)	2 (48)	2 (48)
acquisition time [min:sec]	4:03	4:10	13:34

Abbreviations: GRAPPA = Generalized Autocalibrating Partially Parallel Acquisition; Hz = hertz; HR = high spatial resolution; ms = milliseconds; min = minutes, mm = millimeter; sec = seconds; T = Tesla; TE = echo time; TR = repetition time;° = degree

At 7 T, imaging parameters were modified with the intention to exploit the increased signal-to-noise ratio (SNR) to acquire higher in-plane resolutions and thinner slices for the depiction of DAI while maintaining clinically acceptable scan times compared to the standard sequence at 3 T. Aiming to provide optimal image contrast while maintaining a reasonable scanning time, sequence parameters for SWI from the literature were adapted for this study [15]. The voxel size of the SWI sequences was 0.70 x 0.70 x 2.0 (0.98) mm^3^ at 3 T (interpolated) and 7 T, and 0.25 x 0.25 x 1.5 (0.094) mm^3^ at 7 T with 10-times increased spatial resolution. Detailed protocol parameters are given in Table 2.

### Image Analysis

**Fig 1:**
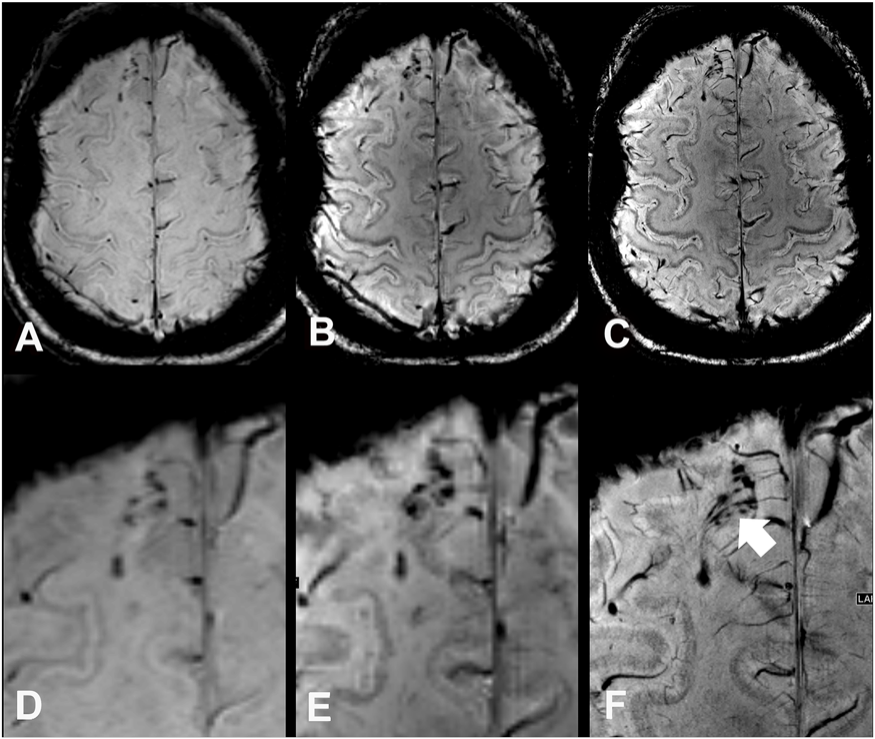
Susceptibility weighted images (SWI) and enlarged regions of interest of a 57 year old female DAI patient (case 6) at 3 T (A, D), at 7 T with equal spatial resolution (B, E), and moreover at 7 T with high spatial resolution (C, F). At 7 T traumatic microbleeds are depicted larger (“blooming effect”), which allows a better discrimination of small lesions and shows a close relation of the traumatic microbleeds to small transcerebral venoles (white arrow). Note the markedly improved grey/white matter contrast in 7 T images when compared to 3T images.

**Fig 2:**
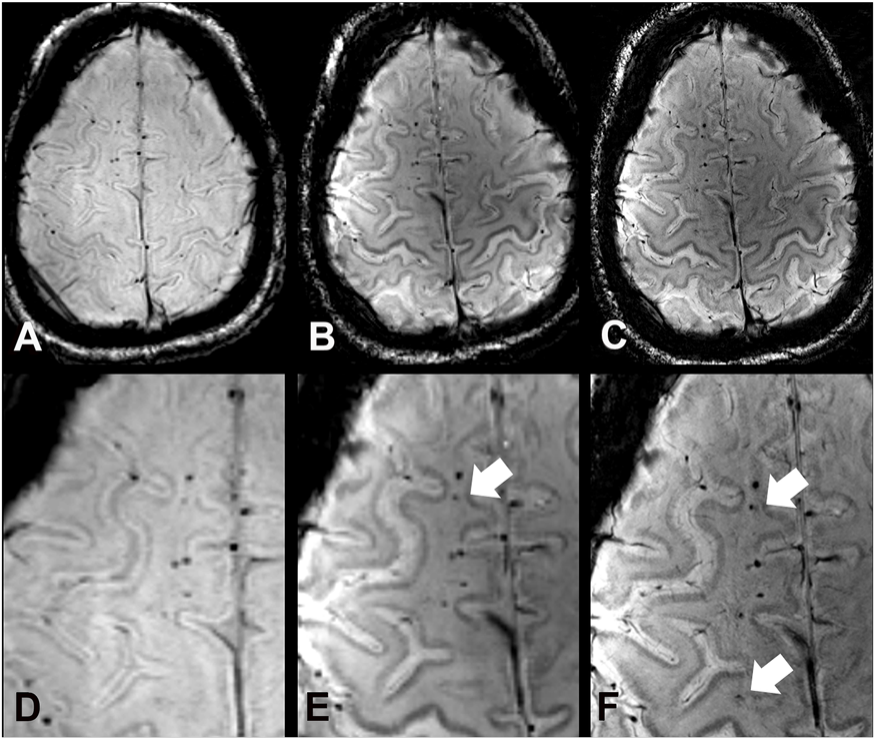
SWI images at 3 T (A), 7 T (B), and 7 T with high spatial resolution (C) of a 44 year old male study participant (case 5), who suffered from a traumatic brain injury in childhood. Images show a line of traumatic microbleeds in the right frontal white matter. Compared to 3 T SW images (A, D) more hemorrhagic DAI lesions are depicted at 7 T with equal (B, E) and higher spatial resolution (C, F) (white arrows).

**Fig 3:**
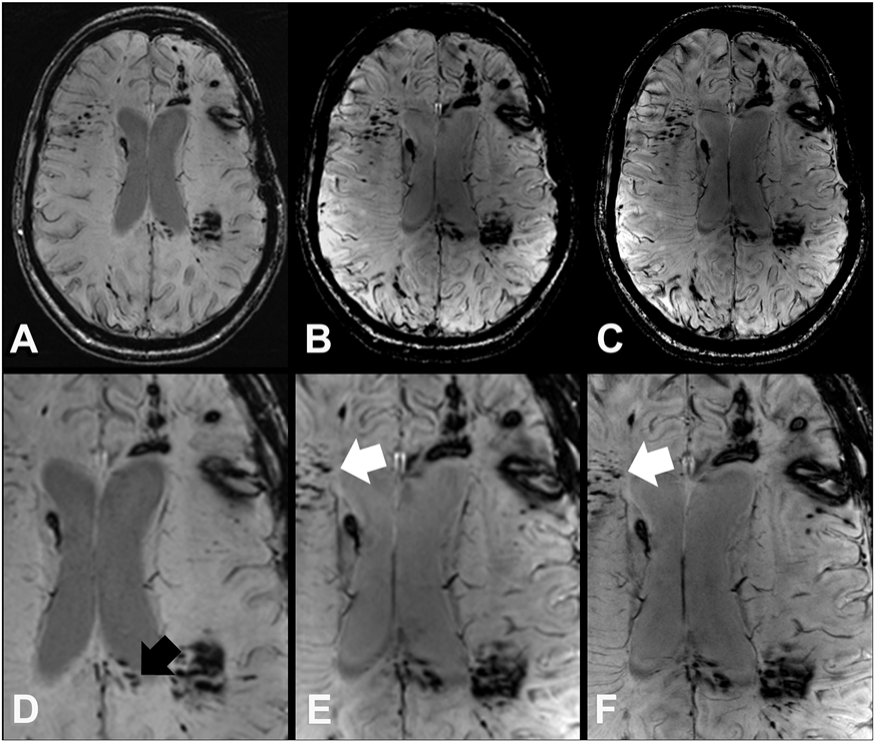
SWI images at 3 T (A, D) 7 T (B, E), and 7 T with high spatial resolution (C, F) of a 25 year old female DAI patient (case 7) survived a polytrauma with acute subdural hematoma, cerebral contusions, and DAI from a car accident with frontal collision. In the splenium (black arrow) more TMB can be discriminated at 3 T SW images (A, D), which merge on 7 T SWI images due to the blooming effect. However, at 7 T SWI images (B, C, E, and F) demonstrate additional TMB in other brain regions like the right frontal white matter (white arrows).

Susceptibility weighted MR images are reconstructed magnitude images that combine the phase information and the magnitude information. The SWI filtered phase data, the magnitude source data, and the merged SWI magnitude data are separately available on our manufacturer´s MR systems. The axial merged SWI magnitude images, which are referred as SWI images in the following, were used to locate and count hemorrhagic DAI. Image analysis was performed by two experienced neuroradiologists (C. M., M. S.) with more than five years´ experience in lesion segmentation. SWI data sets were scored independently by both observers for each study participant. One week after the first reading one observer (C. M.) reevaluated the SWI images of all 10 subjects to assess intraobserver reliability. Both observers reevaluated six scans with a different score in consensus to obtain a final score. Due to the obvious differences in the image appearance between 3 T and 7 T, both observers were not blinded to the field strength and spatial resolution, because the image characteristics inherently revealed this information (Figs. 1–3). Traumatic microbleeds were defined as distinct, rounded or dot-like areas of typical low signal intensity with a diameter of 1 to 10 mm and clear margins on SWI images. If a lesion was larger than 10 mm at 7 T, but not at 3 T, it was included in the TMB count, because 3 T MRI was regarded as clinically established gold standard [21]. Confluent DAI lesions were counted as one lesion on each axial SWI image slice separately. The localization of hemorrhagic DAI lesions was counted separately for eight brain regions, which were defined by the conventional anatomical landmarks: frontal, parietal, temporal and occipital lobes, corpus callosum, brain stem, basal ganglia and cerebellum. The location of DAI lesions was used for the radiological grading of DAI (grade I-III). The diameter of cerebral microbleeds was measured on axial T2*w magnitude source images to exclude effects of the image reconstruction on the lesion size. Twenty-eight reproducible lesions (3 lesions per patient, except from patient 9 with only one detected TMB at both field strengths) were assessed by one observer to compare the intensity of the blooming effect for both field strengths and different sequence parameters (Table 2).

TMBs were distinguished from vascular flow voids (based on sulcal location or linear shape), basal ganglia calcifications, artifacts from adjacent bones or sinuses, or macrohemorrhagic lesions. Calcifications were excluded by using phase information, where calcifications have diamagnetic and TMBs paramagnetic characteristics [22]. Microbleeds close to the skull base and the aerated sinuses were carefully evaluated due to the strong susceptibility effects. All images were reviewed using a Picture Archiving and Communication System (PACS) workstation (General Electric Healthcare, Barrington, IL).

### Statistical analysis

The reliability of the number of microbleeds counted by both observers was quantified by the intraclass correlation coefficient (ICC). The difference in number of identified microbleeds and the diameter of 28 reproducible DAI-associated TMBs was compared between 3 T and both 7 T scans by Wilcoxon matched-pairs signed rank test. A Bonferroni correction was applied to the multiple tests comparing the DAI lesion counts on SWI imagesat 3 T vs. 7 T with similar spatial resolution and 3T vs. 7 T with increased spatial resolution.

Lesion count and lesion diameter were separately compared between:

- 3 T SWI and 7 T SWI,
- 3 T SWI and highly resolved 7 T SWI,
- 7 T SWI and highly resolved 7 T SWI.

All tests were two-sided and a statistically significant difference was considered to be p < 0.05. The analyses were performed by using the statistical software package SPSS (Version 19.0 for Windows; SPSS, Chicago, Illinois).

## Results

After sequence optimization in two healthy volunteers, all 10 study participants with known DAI were successfully examined at both field strengths. None of the volunteers terminated the examination early or reported unexpected side effects during or after the MR exams at 3 T and 7 T. All MR scans were of diagnostic image quality. Minor motion artifacts were seen in patients 1 and 10 in the high resolution SWI at 7 T due to the long acquisition time of over 13 minutes.

### Hemorrhagic DAI count and location

According to the inclusion criteria, hemorrhagic DAI were present in all 10 study participants and diagnosed by both readers (Figs. 1–3).

The number and location of TMB is stated in Table 3 (S1 Dataset).

**Table 3:** Location and count of DAI-associated TMBs in 10 patients.

Field strength	3T SWI			7T SWI			7T SWI HR		
Brain region	n	mean	SD	n	mean	SD	n	mean	SD
frontal lobes	219	21.9	26.7	254	25.4	31.9	281	28.1	31.2
parietal lobes	25	2.5	2.5	39	3.9	5.4	47	4.7	5.3
temporal lobes	113	11.3	15.0	115	11.5	12.6	129	12.9	13.8
occipital lobes	46	4.6	10.0	47	4.7	10.0	80	8.0	16.8
corpus callosum	55	5.5	13.3	92	9.2	23.6	105	10.5	23.9
cerebellum	7	0.7	0.9	7	0.7	1.3	8	0.8	1.4
basal ganglia	8	0.8	1.1	15	1.5	1.9	16	1.6	1.9
brain stem	12	1.2	1.8	13	1.3	1.6	18	1.8	2.5
whole brain	485	48.5	57.2	584	58.4	77.1	684	68.4	84.1

Results showing the amount of counted TMBs after consensus of both observers in 10 DAI patients. HR = high resolution; median = median value; n = number of traumatic microbleeds; SD = standard deviation; SWI = susceptibility weighted imaging.

In the whole brain, comparative imaging revealed a total of 485 TMBs (range 1–190, median 25) at 3 T, 584 TMBs (plus 20%, range 1–262, median 30.5) at 7 T with equal spatial resolution, and 684 TMBs (plus 41%, range 1–288, median 39.5) at 7 T with increased spatial resolution. 7 T SWI depicted significantly more TMB in the frontal lobes (plus 15.9%, range 0–109, median 18; Figs. 1, 2) compared to 3 T with similar spatial resolution. A 10-times higher spatial resolution in terms of voxel volume at 7 T even allowed the depiction of significantly more TMB in the corpus callosum (plus 87.6%, range 0–77, median 1), frontal (plus 28.3%, range 0–108, median 20.5) and temporal lobes (plus 14.1%, range 0–42, median 6.5) compared to the clinical 3 T SWI sequence (Fig. 1). The higher spatial resolution of 7 T SWI was beneficial for the significantly improved depiction of additional TMB in the frontal, temporal, and occipital lobes (Table 4, Fig. 3).

**Table 4:** Hemorrhagic DAI lesion counts compared between 3T and 7T SWI with equal and high spatial resolution by Wilcoxon signed-rank test.

MR sequences	3T SWI/ 7T SWI	3T SWI/7T SWI HR	7TSWI/7T SWI HR
Brain regions	Wilcoxon test after Bonferroni correction p	Wilcoxon test after Bonferroni correction p	Wilcoxon test p
frontal	0.056	0.016*	0.044*
parietal	1.000	0.242	0.072
temporal	0.262	0.052	0.023*
occipital	1.000	0.136	0.042*
corpus callosum	0.132	0.084	0.066
cerebellum	1.000	1.000	0.655
basal ganglia	0.204	0.132	0.317
brainstem	1.000	0.570	0.414
whole brain	0.042*	0.016*	0.015*

* = Statistically significant values (p > 0.05).

On high resolution 7 T SWI images an equal or increased number of DAI lesions was counted in all brain regions compared to the lower resolved images, which was referred to the reduced slice thickness and partial volume effect. In functional important and anatomical small areas like the brainstem and the corpus callosum a small increase of the lesion count was apparent with increasing field strength and spatial resolution, although it was not statistically significant (Table 3 and Table 4).

### TMB size

The diameters of 28 reproducible hemorrhagic DAI lesions of 10 patients were compared between SWI images at 3 T and 7 T with equal and increased spatial resolution (Table 5, S2 Dataset).

**Table 5:** Comparison of quantity and size of DAI-associated TMBs between field strengths.

Parameter	3T SWI	7T SWI	7T SWI HR
TMB count	485	584	684
TMB mean size [mm]	3.017	3.937	3.993
SD [mm]	1.495	1.753	1.709

Results showing the amount of counted TMBs after consensus of both observers and the varying sizes in up to 3 reproducible lesions in each of the 10 DAI patients (except from patient 9 with only one TMB).

DAI lesions were depicted significantly larger at 7 T SWI images with standard (r_s_ = 0.984; p = 0.005) and increased, non-clinical spatial resolution (r_s_ = 0.967; p = 0.007) compared to the 3 T SWI images. The diameter of DAI lesions measured on 7 T SWI images with different spatial resolutions was almost equal (r_s_ = 0.964; p = 0.776).

### Interobserver and intraobserver reliability

For the number of DAI-associated microbleeds, high interobserver reliability was demonstrated for the 3 T SWI scan (ICC = 0.998, 95% CI = 0.990–0.999), the 7 T SWI scan with equal spatial resolution (ICC = 1.0; 95% CI = 0.999–1.0), and for the 7 T SWI scan with higher spatial resolution (ICC = 0.993; 95% CI = 0.968–0.998) compared to 3 T. Intraobserver reliability for the number of visible TMBs was excellent for the 3 T SWI scan (ICC = 1.0, 95% CI = 0.999–1.0), the 7 T SWI scan with equal spatial resolution (ICC = 1.0, 95% CI = 0.999–1.0), and for the 7 T SWI scan with higher spatial resolution (ICC = 0.998; 95% CI = 0.989–1.0) compared to 3 T.

## Discussion

Diffuse axonal injury can be diagnosed by clinical and radiological signs [23,24]. The most important diagnostic step is to think of DAI, if TBI patients are symptomatic with posttraumatic coma, neurological deficits and cognitive impairment, although trauma head CT appears normal [12]. MRI is the imaging modality of choice to detect hemorrhagic and non-hemorrhagic DAI, because CT exams are often false negative towards DAI [12]. Currently T2*w GRE sequences and increasingly SWI are used for the detection of cerebral microbleeds as radiological hallmark of DAI [10,14,18,25]. Non-hemorrhagic DAI, based on a widespread disruption of axons, can be diagnosed with diffusion tensor imaging (DTI), diffusion weighted imaging (DWI), MR spectroscopy, and conventional MR sequences [24,26–28].

The sensitivity of MRI for susceptibility contrasts increases almost linear with the magnetic field strengths, which is beneficial for the depiction of DAI with SWI [29–32]. Susceptibility weighted imaging is up to six times more sensitive than T2*w GRE sequences to paramagnetic substances like hemosiderin [10,13,18,33]. Since first description in 1997, SWI is increasingly applied in clinical routine for the depiction of cerebral microbleeds, calcifications, and venous vasculature [15,34,35]. The better conspicuity of small microbleeds at higher field strengths is based on several factors like higher achievable spatial resolution with thinner sections, enhanced susceptibility effect, pronounced blooming effect, better differentiation from small venous vessels, and higher signal-to-noise ratio as reported from previous studies [13,17,18,21]. Microbleeds appear larger on 7 T SWI images when compared to images acquired at lower field strengths [13,18]. The so called “blooming effect” causes earlier dephasing of the surrounding tissue signal and increases with higher field strengths and longer TE [14,36]. On the one hand this effect additionally improves the depiction of very small TMB and venous vessels, which thus become detectable at all. On the other hand, adjacent microbleeds merge with each other at higher field strengths, which reduces discriminability and the number of countable lesions. As a partial solution thinner sections may reduce partial volume effects and overlaps of deoxyhemoglobin containing veins, focal calcifications or ferritin-containing parts of the basal ganglia and the cerebellar nuclei [20,21]. The diagnostic potential of SWI for the depiction of non-traumatic microbleeds has already been described at 3 T or 7 T MRI in patients with cerebral amyloid angiopathy, vascular dementia, cerebrovascular disease and antithrombotic medication [10,13,18,34,37,38]. The prevalence of TMB is dependent on the incidence of TBI [1]. In a prospective cohort study including 159 patients after moderate to severe head trauma DAI were detected in 72% by 1.5 T MRI [2]. In 20 patients with mild head trauma the prevalence of MR signs for hemorrhagic DAI was 20%, although CT scans were normal [12].

To our best knowledge, the diagnostic benefit of 7 T SWI has not been evaluated for TMB until now. However, a 7 T MR spectroscopy imaging (MRSI) case-control study including 25 veterans with mild traumatic brain injury and memory impairment revealed hippocampal injury by significantly decreased N-acetyl aspartate to choline and N-acetyl aspartate to creatine compared to 20 healthy controls [39]. In the present study including 10 DAI patients, two observers diagnosed 20% more TBI on 7 T SWI images with similar resolution and 41% more TBI on 7 T SWI images with 10-fold higher resolution compared to clinical 3 T SWI images. Inter- and intraoberser reliability for counted TMB was very high at both field strengths, but slightly higher at 7 T with similar resolution. These expected findings are in line with comparison studies between 1.5 T and 7 T [18,21]. Although the field strength was more than doubled from 3 T to 7 T in this study, the TMB count did not increase in equal manner. This fact implies that 1.5 T and moreover 3 T SWI images are already suitable to diagnose hemorrhagic DAI in the vast majority of cases [10].

This raises the question of a diagnostic benefit for patients with probable DAI to be examined at 7 T.

A diagnostic problem is present for patients with chronic neurological, neuropsychological, and cognitive impairment after head trauma e. g. post-concussion syndrome, who lack evidence of structural brain damage on routine MRI [40]. Data of patients with histopathologically confirmed DAI and false-negative routine MRI are not available. A retrospective analysis of 3 T MRI examinations, including 274 patients with a primary diagnosis of TBI, revealed TMB in 148 (46%) patients and no chronic signs of traumatic tissue alterations in 76 patients (23.8%)[3]. In the same study population a plain relationship between traumatic structural and functional brain alterations could not be revealed after comparing neuroradiological findings and neuropsychological exams. This problem is intrinsically related to the diffuse nature of TBI and cannot be solved by simply increasing the sensitivity for detecting mircrobleeds. However, an improved detection of cerebral microbleeds in functionally strategic localizations e.g. corpus callosum and brainstem is important for the grading of DAI patients. Additionally, the increased sensitivity of UHF MRI for hemorrhagic DAI can be expected to facilitate the diagnosis of isolated DAI in cases with low burden of TMB. Although TMB are known to be detectable for years, their visibility correlates negatively with the time interval from trauma [41,42]. On condition that 7 T MRI will be approved officially for clinical examinations in future, patients without evidence of TBI at 3 T, but with cognitive impairment and other neuropsychologic disorders after head trauma may profit from 7 T MRI. The improved lesion detection may be relevant for medical assessment in medicolegal cases that need either to confirm or to rule out the diagnosis of DAI years after TBI.

As a consequence prospective longitudinal studies including more TBI patients and MR exams at different (ultra-) high field strengths are needed for clarification. The cost-benefit analysis of UHF MRI is dependent on further clinical application studies, which may allow transforming this research instrument into a clinical diagnostic tool in future.

Limitations of our study are based on the small number of study participants with DAI, the retrospective enrollment after a variable time span after trauma, and the differences of sequence parameters between both field strengths. MR sequence protocols were adapted to both field strengths to ensure high diagnostic image quality and spatial resolution in a reasonable scan time. Under these circumstances it was not possible to adapt scanner and sequence parameters at 3 T and 7 T with the main magnetic field strength being the only variable difference between the two scanning modalities. A direct signal-to-noise ratio (SNR) or contrast-to-noise ratio (CNR) comparison was not possible since parallel imaging was used at both field strengths. In the scientific setting diffusion tensor imaging (DTI) and DTI fiber tractography have shown to be sensitive and useful methods to visualize and quantify DAI-related white matter lesions at field strengths up to 3 T [27,43,44]. Preliminary *in vivo* and *ex vivo* studies have demonstrated the feasibility of 7 T DTI [45–47]. But this method was not implemented in this study in order to keep the total examination time reasonable as well as due to hardware constraints on the gradient amplitude.

## Conclusion

In conclusion, this study reveals that 7 T SWI is significantly more sensitive than 3 T SWI for the depiction of hemorrhagic DAI. Hence, UHF MRI at 7 T may become a complementary diagnostic tool to 1.5 T and 3 T MRI for the diagnosis of DAI in inconclusive or in medicolegal cases. Larger prospective studies are needed to prove the diagnostic benefit of UHF MRI for head trauma patients suspicious of DAI, despite normal findings on conventional MRI.

## Supporting Information

S1 Dataset Source data of counted traumatic microbleeds in 10 patients with diffuse axonal injury on SWI images at 3T and 7T (with equal and higher spatial resolution) by two readers. Legend: SWI = susceptibility weighted imaging, 3T = 3 Tesla MRI, 7T = 7 Tesla MRI, TMB = traumatic cerebral microbleeds
(XLSX)

S2 Dataset Measured diameters of three corresponding traumatic microbleeds on SWI images at 3T and 7T in 10 DAI patients (in mm). Legend: ID = identification, TMB = traumatic microbleed, mean = mean value, SD = standrad deviation
(XLSX)
